# The gram-negative bacterial periplasm: Size matters

**DOI:** 10.1371/journal.pbio.2004935

**Published:** 2018-01-17

**Authors:** Samuel I. Miller, Nina R. Salama

**Affiliations:** 1 Departments of Genome Sciences, University of Washington, Seattle, Washington, United States of America; 2 Department of Medicine, University of Washington, Seattle, Washington, United States of America; 3 Department of Microbiology, University of Washington, Seattle, Washington, United States of America; 4 Human Biology Division, Fred Hutchinson Cancer Research Center, Seattle, Washington, United States of America

## Abstract

Gram-negative bacteria are surrounded by two membrane bilayers separated by a space termed the periplasm. The periplasm is a multipurpose compartment separate from the cytoplasm whose distinct reducing environment allows more efficient and diverse mechanisms of protein oxidation, folding, and quality control. The periplasm also contains structural elements and important environmental sensing modules, and it allows complex nanomachines to span the cell envelope. Recent work indicates that the size or intermembrane distance of the periplasm is controlled by periplasmic lipoproteins that anchor the outer membrane to the periplasmic peptidoglycan polymer. This periplasm intermembrane distance is critical for sensing outer membrane damage and dictates length of the flagellar periplasmic rotor, which controls motility. These exciting results resolve longstanding debates about whether the periplasmic distance has a biological function and raise the possibility that the mechanisms for maintenance of periplasmic size could be exploited for antibiotic development.

Gram-negative bacteria, like the energy organelles of plants and animals (the chloroplast and mitochondria), have two membrane bilayers termed the outer and inner membranes. The space between these two membranes is termed the periplasm. Long before single-cell eukaryotes, the periplasm evolved as the first extracytoplasmic compartment to provide an important competitive adaption to gram-negative bacteria. Early knowledge and the discovery of the periplasm developed even before its morphological visualization. In the 1960s, scientists were trying to understand how toxic enzymes involved in degradation of important biological molecules, such as ribonucleases and phosphatases produced by the gram-negative bacteria *Escherichia coli*, were not toxic to the cell. Biochemical extraction methods suggested a separate compartment, because such extraction preserved the inner membrane-bound cytoplasm, and these spheroplasts could grow again and synthesize more enzymes [1]. The development of electron microscopy led to the visualization of the two membrane bilayers separated by the periplasm [2].

The additional membrane allows for the creation of the periplasm as a separate cellular compartment whose novel functions likely provided a significant and perhaps even more important selective advantage than toxin exclusion (Table 1). These novel functions include protein transport, folding, oxidation, and quality control similar to the eukaryotic cell endoplasmic reticulum. The periplasm also allows for the sequestration of enzymes that may be toxic in the cytoplasm, important signaling functions, and cell division regulation. Additionally, it contributes to the ability of the cell to withstand turgor pressure by providing structural systems that work in concert with the outer membrane, such as peptidoglycan and lipoproteins, multidrug efflux systems, and specific solutes that contribute to a Donnan or ionic potential across the outer membrane. The periplasm also contains the assembly platforms involved in secretion of uniquely structured beta-barrel proteins, lipoproteins, and glycerolphospholipids to the outer membrane (Fig 1).

**Table 1:** Functions of the periplasm [20].

Protein oxidation
Protein secretion
Protein folding
Lipopolysaccharide secretion to the outer membrane
Lipoprotein secretion
Proteases
Phosphatases
Nucleases
Phospholipases
Environmental sensing
Maintenance of the Donnan potential across the outer membrane
Peptidoglycan synthesis
Cell division machinery
Envelope stress responses
ABC transporter
Flagellar rotor
Nitrate reduction
Biosynthesis of molybdenum and its incorporation into enzymes
Electron transport
Iron and metal transport
Osmoregulation
Sensing and resistance to cationic antimicrobial peptides

Abbreviation: ABC, ATP-binding cassette.

**Fig 1:**
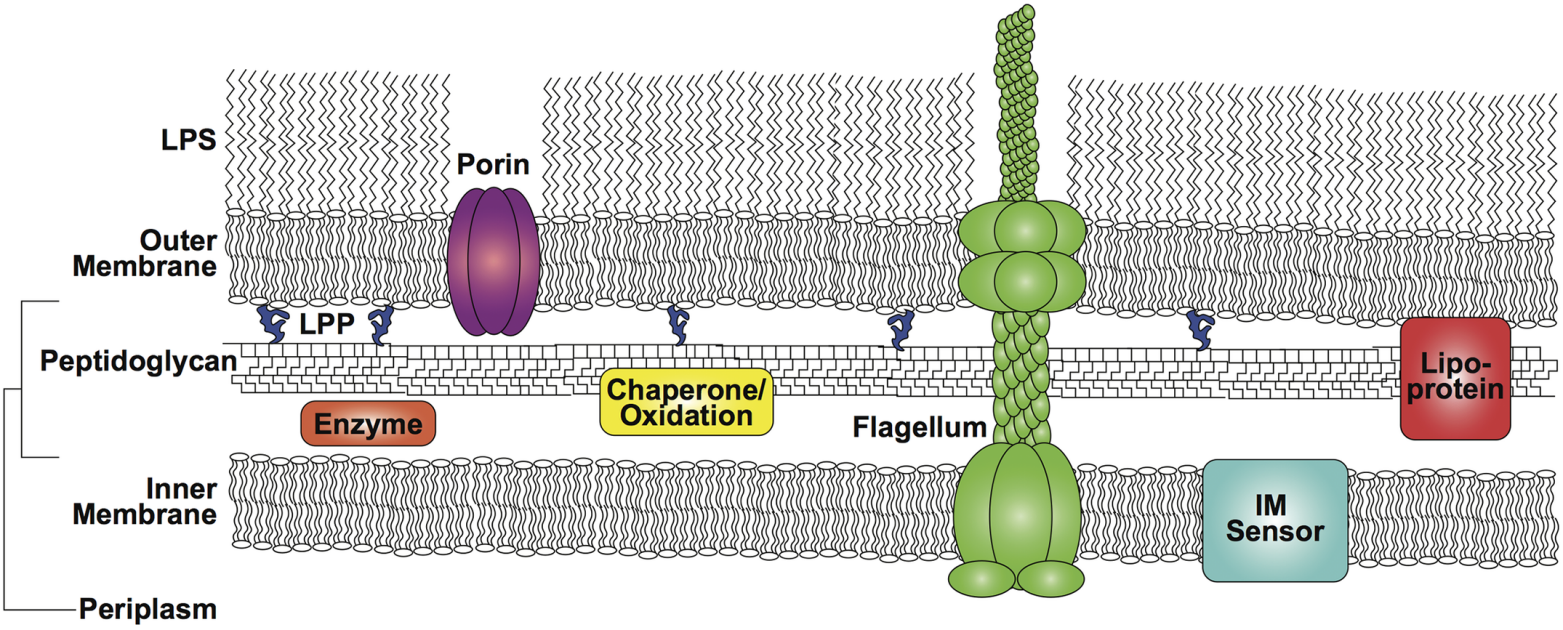
Architecture of the gram-negative bacterial cell envelope. Shown is the asymmetric bilayer of lipopolysaccharide and glycerolphospholipids that comprise the outer membrane. The inner membrane is a symmetric bilayer of glycerolphospholipids. The periplasmic space is the region between these membranes that includes a variety of enzymes and functions, including the oxidation and quality control of proteins. Also within the periplasmic space is a layer of crosslinked sugars and amino acids termed peptidoglycan, which surrounds the cell. The peptidoglycan is linked to the outer membrane in enteric bacteria through covalent transpeptidase linkages between an abundant outer membrane lipoprotein Lpp. A variety of sensors sit in the inner membrane with periplasmic domains sensing environmental change and, in the case of the Rcs system, a change in location of the RcsF outer membrane lipoprotein. Multicomponent protein complexes such as the flagellar machine span the two membranes. IM, inner membrane; Lpp, Braun’s lipoprotein; LPS, lipopolysaccharide; RcsF, Regulator of capsule synthesis F.

The outer membrane is a unique organelle connected to other parts of the cell envelope via the periplasm. Gram-positive bacteria lack an outer membrane but have a more extensive peptidoglycan polymer protecting their surface. In contrast to the bacterial inner membrane—which is a bilayer of glycerolphospholipids similar to that of most mammalian membranes and which has specific flow characterized by lateral diffusion—the outer membrane has restricted flow [3]. It is a unique bilayer, with the inner leaflet having a typical glycerolphospholipid content of phosphotidylethanolamine, phosphatidylglycerol, and cardiolipin and the outer leaflet largely composed of a unique glycolipid, lipopolysaccharide (LPS) [4]. The LPS phosphates confer a negative charge to the surface, and a specific Donnan potential is created across the outer membrane into the periplasm [5]. The outer membrane functions as a selective barrier that allows the transport of valuable nutrients while providing a barrier against toxic compounds, such as cationic antimicrobial compounds produced by all organisms, including many gram-positive bacteria [6]. Another component of this barrier are outer membrane proteins with a unique beta-barrel structure that are inserted into the outer membrane through a specific periplasmic chaperone system [7]. These proteins assemble into the outer membrane as specific puncta, indicating the outer membrane likely assembles into specific discrete patches containing protein and the unique asymmetric lipid bilayer [8]. Included among these outer membrane proteins are the porins, which can act as selective channels that allow hydrophilic substrates of a specific size entrance to the periplasm. Luckily for humans, these porins transport hydrophilic beta-lactam antibiotics, which allows their penetration into the periplasm, where they target the synthesis of the important structural element of the cell wall—the polymeric peptidoglycan. The outer membrane in some bacteria is anchored to the peptidoglycan polymer through abundant lipoproteins, which are inserted into the inner leaflet of the outer membrane through specific secretion systems [9]. A variety of important protein complexes function as nanomachines and utilize ATP hydrolysis to secrete macromolecules or turn a motility organelle termed the flagella [10,11,12]. Therefore, the outer membrane and the inner membrane are also connected across the periplasm by membrane-spanning protein complexes. Hence, the outer membrane is composed of distinctly assembled patches that comprise a complex organelle that can be attached to the peptidoglycan layer and the inner membrane through covalent and noncovalent protein linkages. The assembly of the outer membrane and its link to the peptidoglycan and cytoplasm creates a space between the inner membrane and the outer membrane, which is the periplasm.

Despite the important functions contained within the periplasmic space, for many years there has been debate about the intermembrane distance or size of this compartment and whether there is uniformity of spacing between the inner and outer membranes throughout the cell. There was concern that many of the visualizations of this space as being of a specific size were artifacts of fixation for imaging by electron microscopy and that, in fact, the space was actually only a potential space. The early electron microscopic studies of Bayer demonstrated adhesions between the outer and inner membrane that obliterated part of these spaces; he suggested that points of adhesion were areas where the major outer leaflet lipid, LPS, was delivered to the outer membrane from its site of synthesis at the inner membrane [13]. However, his work was subsequently discredited as being derived from observation of potential fixation artifacts, though many experts today think that there may be real protein-based adhesions between the membranes because some efflux and transport systems do not contain components of sufficient dimensions to span the visualized space. The presence of specific areas in which the membranes are close together would explain how some of these ATP-binding cassette (ABC) transport and efflux pumps could work; these systems have periplasmic protein components that are essential for efflux, LPS, or other glycolipid transport but lack an intrinsic size or polymeric nature large enough to reach the outer membrane and thus provide a mechanism to promote transport. Furthermore, the periplasm contains many other components that necessitate at least some volume for the periplasmic space, most prominently the peptidoglycan polymeric layer surrounding the cell. At present, it is unclear how these transporters get around this polymer and the width of the periplasm to contact the membrane, though recent work demonstrating that outer membrane lipoproteins can coordinate peptidoglycan synthesis through direct contact indicates that at least some proteins may fit through pores in peptidoglycan to accomplish important functions [14]

In contrast, a variety of organelles, including the flagellum and the virulence-associated Type III secretion system needle complex, require the assembly of polymers within the periplasm that span the two membranes. In the case of the flagellum, its rod or driveshaft spans the periplasm, and its length is determined by the polymer contacting the outer membrane. Elegant recent work by the group of Kelly Hughes has shown that the size of the periplasm, or the distance between the two membranes, is controlled largely in enteric bacteria by a specific lipoprotein termed Braun’s lipoprotein (or Lpp), which covalently links the outer membrane to the peptidoglycan layer [15]. This is quite remarkable because Lpp is the most abundant protein present in enteric bacteria, described by Braun 48 years ago, and until this point no specific function had been ascribed to it. This alpha-helical protein is inserted through its lipid anchor into the inner leaflet of the outer membrane and covalently linked to the peptidoglycan polymer by a family of transpeptidases [16]. Lengthening these lipoproteins that allow expansion of the periplasm leads to a longer flagellar rod and more efficient swimming behavior. These authors interpreted this result as indicating that there must be other evolutionarily selected functions that limited the periplasmic size, forcing a reduction in swimming efficiency. In this issue of *PLOS Biology*, one of those important functions is revealed: a signaling function of envelope damage controlled by another outer membrane lipoprotein, Regulator of capsule synthesis F (RcsF), which senses disorder or damage of the envelope.

Gram-negative bacteria have a variety of important functions that sense membrane damage and toxic compounds, such as antimicrobial peptides, which damage the outer membrane [17,18]. These sensing systems include those that allow remodeling of the bacterial surface to be more resistant to toxic compounds—analogous to spaceships energizing their shields in science fiction stories [19]. Some of these sensing systems are receptors that function as sensor kinases with domains in the periplasm to sense specific molecules or damage. However, one of the more unique sensor kinase systems, termed the Rcs system—which on membrane damage activates synthesis of extracellular polysaccharide to provide cellular protection and biofilm formation—has an outer membrane lipoprotein RcsF, which interacts with signaling proteins with specific periplasmic domains on envelope damage and peptidoglycan stress to activate the synthesis of extracellular polysaccharide production and other stress-related coping pathways [20]. Thus, envelope damage in some way brings the RcsF lipoprotein in greater proximity to the inner membrane-sensing system, and thus it evolved to sense disorder in the outer membrane and/or peptidoglycan (Fig 2). In this issue of *PLOS Biology*, the authors conclusively demonstrate that this sensing requires the periplasm to be a specific size because mutations that lengthen the highly abundant Lpp lipoprotein anchor from the outer membrane to the peptidoglycan (resulting in an increased size of the periplasm) abolished signaling unless the sensing lipoprotein (which on membrane damage must reach to the inner membrane sensor) is also lengthened [21]. This work also clearly shows a very specific order and size to the periplasm; the size of the periplasm is clearly seen as it exists in association with the changes in lipoprotein anchoring or length by cryo-electron microscopy. This technology and electron tomography used in the work of the Hughes group in relation to the flagellar rotor [15] are revolutionizing our view of the bacterial cell envelope and the protein complexes that span the periplasm to perform important functions [22].

**Fig 2:**
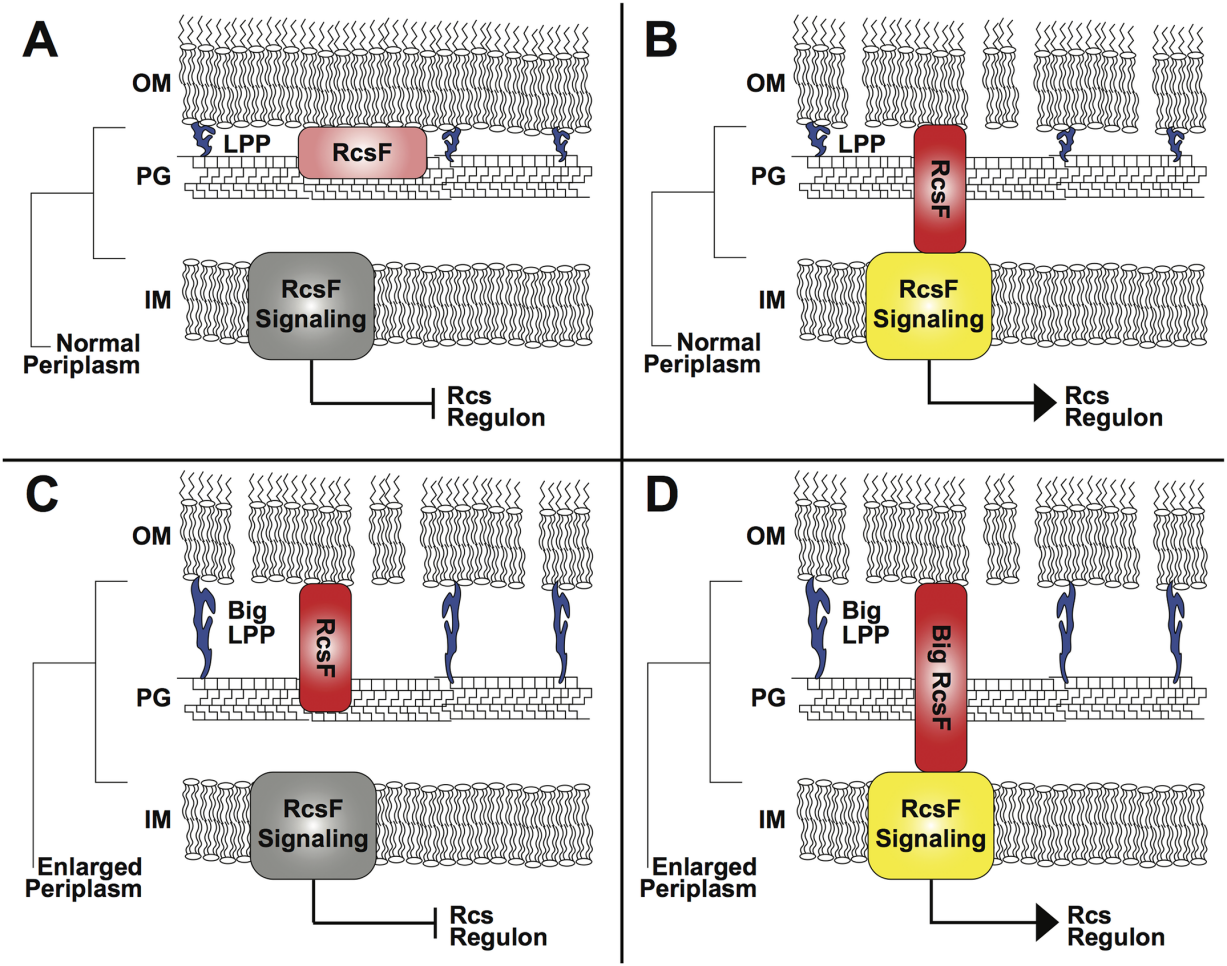
RcsF signaling is altered by a change in size of the periplasmic space. The RcsF outer membrane lipoprotein sensor must contact its inner membrane signaling partners to activate sensing. This sensing requires a specific periplasmic distance because lengthening of the Lpp linkages to peptidoglycan increases the distance of the periplasm, and unless RcsF is lengthened, signaling can no longer occur. In panel A: the state in which RcsF is not activating signaling because no envelope disorder is ongoing. In panel B: envelope disorder leads to RcsF physical interactions with the inner membrane-sensing system, and the Rcs regulon is activated. In panel C, in which Lpp has been lengthened and the periplasmic intermembrane distance lengthened, the Rcs regulon cannot be activated despite envelope disorder. In panel D: the defect of the long Lpp is corrected by lengthening RcsF. IM, inner membrane; Lpp, Braun’s lipoprotein; OM, outer membrane; PG, peptidoglycan; RcsF, Regulator of capsule synthesis F.

Though these recent studies have defined Lpp as a specific molecular ruler between the outer membrane and peptidoglycan, it is unknown what regulates the distance between the inner membrane and peptidoglycan and what controls the polymerization or degradation of the peptidoglycan polymer so that it does not fully obstruct proteins that span the periplasm. Defining these and other mysteries of the cell envelope could lead to important practical advances in addition to satisfying our scientific drive to solve the mysteries of the gram-negative bacterial cell envelope. This envelope is a remarkably efficient and evolutionarily advanced molecular sieve that makes the development of antibiotics against these organisms much more difficult than for gram-positive bacteria, which lack the additional membrane and periplasm.

Increased knowledge of gram-negative cell envelope is also critical for understanding the mechanisms of antibiotic resistance because many of our most successful antibiotics, including beta-lactam antibiotics (which target peptidoglycan and enter through the porins), target the cell envelope. Gram-negative bacteria and multidrug-resistant organisms continue to evolve through envelope mutations and the acquisition of new periplasmic enzymes. There is a lack of new antibiotics for gram-negative bacteria in the pipeline because of the difficulty of breaching the unique barrier provided by the outer membrane and the periplasm. In this regard, antibiotics with periplasmic targets have an advantage over those faced with the difficulties of penetrating the inner membrane and avoiding significant efflux. It is interesting to speculate that targeting essential periplasmic functions that require a periplasm of specific size and ability to accommodate different functions could offer important new objectives for antibiotic development. Recent studies have uncovered new basic functions of the gram-negative envelope through bacterial genetics, structural biology, and advanced morphological techniques. Despite decades of study, much remains to be learned about the gram-negative bacterial cell envelope. Uncovering other mysteries in this area should lead to a new generation of targets for the development of antibiotics to keep us one step ahead in the arms race with antibiotic-resistant gram-negative bacteria.

## Abbreviations

ABC: ATP-binding cassette
IM: inner membrane
Lpp: Braun’s lipoprotein
LPS: lipopolysaccharide
OM: outer membrane
PG: peptidoglycan
RcsF: Regulator of capsule synthesis F
